# TC2-Res: a structured fusion of tract-level and connectome-level brain imaging in small-sample cohorts of athletes

**DOI:** 10.3389/fnana.2026.1841420

**Published:** 2026-06-03

**Authors:** Yang Shi, Lixian Chen, Mingxuan Huang, Zhiqi Huang, Jiayu Ou, Junwei Zeng

**Affiliations:** 1 School of Computer Science and Technology, Guangdong University of Technology, Guangzhou, China; 2 School of Management, Guangdong University of Technology, Guangzhou, China; 3 Zhongshan School of Medicine, Sun Yat-sen University, Guangzhou, China; 4 School of Physical Education, Guangdong University of Technology, Guangzhou, China

**Keywords:** athlete, brain imaging, connectome, consistency regularization, small-sample, structural fusion, tract-level imaging

## Abstract

Combining diffusion tract descriptors with structural connectome descriptors may help characterize athlete-related brain imaging patterns; however, this approach is challenging in small-sample studies, where flexible learned models can easily overfit. In such settings, simple linear classifiers often serve as strong baselines, although they do not explicitly encode the anatomical correspondence between tract-level microstructural descriptors and connectome-level organization. To address this gap, we introduce Tensor Connectome Consistency Residual (TC2-Res), a lightweight structured fusion framework that combines pair-aware tract representations, global modality branches, family-level encoders, and a tract-connectome consistency regularizer that encourages matched anatomical families to align in a shared latent space. In a small cohort of collegiate athletes, TC2-Res achieved a slightly higher mean balanced accuracy compared to a matched naive learned fusion baseline on the primary football-vs.-others task, whereas a classical linear support vector machine (SVM) remained the strongest overall classifier. The observed performance gain was modest and inconsistent across folds, and evidence of improvement specifically attributable to the consistency term was limited. These results suggest that anatomically structured fusion represents a plausible lightweight design direction for learned multimodal classification in limited-data settings while also highlighting the continued strength of classical linear baselines in this regime.

## 1 Introduction

Brain imaging studies often analyze both local white matter microstructure and global structural connectivity to characterize subtle differences in brain phenotypes, including those observed in athlete populations (Zhang et al., 2022; Caron et al., 2021; Goubran et al., 2023). However, integrating these two sources of information remains difficult when the sample is small and the feature dimension is high (Varoquaux, 2018; Varoquaux and Cheplygina, 2022). Existing fusion pipelines typically treat tract-level and connectome-level descriptors as separate inputs and do not explicitly model the anatomical correspondence (Zhang et al., 2020; Schiavi et al., 2020) between them. This creates a practical modeling dilemma. Simple linear models often remain competitive under limited-data conditions (Schulz et al., 2020; Varoquaux and Cheplygina, 2022), but they lack an explicit mechanism for coupling the microstructural evidence of anatomically defined tracts with the topological evidence of the structural connectome.

Recent multimodal neuroimaging studies have explored early fusion, late fusion, and regularized representation learning (Zhang et al., 2020; Luo et al., 2024). Nevertheless, two issues remain particularly important in small-sample brain phenotyping (Varoquaux, 2018; Varoquaux and Cheplygina, 2022). First, naive learned fusion models can expend their capacity on unstable cross-modal interactions that are only weakly supported by a small training cohort (Varoquaux, 2018; Varoquaux and Cheplygina, 2022). Second, treating tract descriptors and connectome descriptors as independent variables ignores the fact that both modalities reflect the same anatomical organization at different scales (Zhang et al., 2022; Schiavi et al., 2020; Beer et al., 2019). Features that belong to the same anatomical family should not evolve independently; structural constraints (Beer et al., 2019; Ngiam et al., 2011) are necessary. Therefore, an effective learned model for this setting should remain lightweight while directly embedding anatomical structure into the representation (Varoquaux, 2018; Schulz et al., 2020; Beer et al., 2019). In addition, it should include an explicit mechanism that encourages agreement between the matched tract and connectome evidence.

In this study, we investigate whether anatomically structured fusion can improve the learned multimodal classification in a severely small-sample setting. Rather than aiming to outperform every classical baseline, we focus on a narrower question—Can a lightweight learned fusion model that explicitly encodes tract-connectome correspondence improve upon naive learned fusion while remaining interpretable and statistically restrained? To this end, we introduce Tensor Connectome Consistency Residual (TC2-Res), which combines pair-aware tract summarization, family-aligned tract and graph encoders, global modality branches, and a mild tract-connectome consistency constraint.

Our empirical results are mixed. In the primary football-vs.-others task, TC2-Res is the strongest structured learned fusion variant among the configurations tested and achieves a small positive mean difference relative to a matched naive learned fusion baseline. However, this advantage is heterogeneous across folds, and a classical linear support vector machine remains the strongest overall classifier. In the secondary three-class task, TC2-style models do not provide the strongest learned result. Therefore, we treat the method as a lightweight structured fusion approach with task- and comparison-dependent benefits rather than as a uniformly superior classifier.

**Figure 1:**
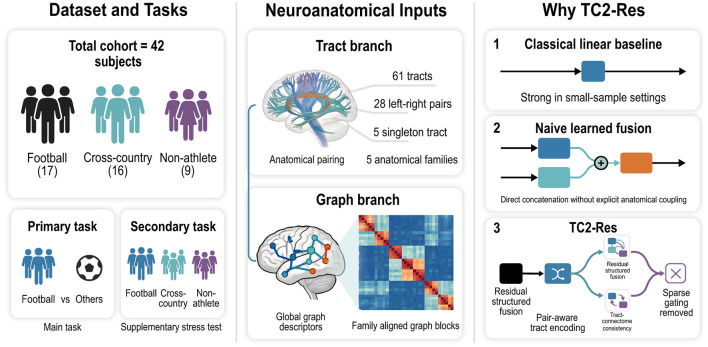
Overview of the study design, neuroanatomical inputs, and the TC2-Res fusion framework. **(Left)** The dataset consists of a small cohort of 42 collegiate subjects, divided into a primary binary classification task (football athletes vs. others) and a secondary three-class robustness task. **(Middle)** The multimodal inputs include diffusion tractography (pair-aware tracts grouped into five anatomical families) and structural connectomics (global and family-aligned graph descriptors). **(Right)** Motivation for TC2-Res: While classical linear baselines are stable in small-sample regimes and naive learned fusion fails to capture the anatomical correspondence, TC2-Res addresses this gap by utilizing retained global modality branches, pair-aware encoding, and a family-level tract-connectome consistency regularizer.

The overall study design and motivation for TC2-Res are summarized in Figure 1.

The contributions of this study are as follows.

1. We propose a lightweight anatomically structured fusion framework for combining tract-level and connectome-level brain imaging descriptors through pair-aware tract summarization, family-level encoders, and retained global modality branches.
2. We introduce a tract-connectome consistency regularizer that aligns matched anatomical families in a shared latent space and evaluate its empirical contribution under matched ablations.
3. We provide an explicit formulation of the pair-aware tract representation and clarify the disagreement magnitude controlled by the family-aligned consistency term.
4. We report both positive and negative findings on a small cohort of collegiate athletes, showing a modest aggregate advantage over naive learned fusion for the primary task while also demonstrating that classical linear baselines remain strongest overall and that the same inductive bias does not transfer robustly to the secondary task.

## 2 Related studies

### 2.1 Brain imaging classification in the small-sample regime

Classification of brain imaging data in the small-sample regime remains difficult because the number of subjects is often far lower than the dimensions of the tract or connectome features (Thomas et al., 2019; Nenning and Langs, 2022). In this setting, strong linear or kernel methods can achieve competitive or superior performance because the effective complexity of these methods is better controlled than that of flexible deep learning models (Schulz et al., 2020; Song et al., 2016). This empirical pattern appears repeatedly in studies of diffusion imaging, connectomics, and general neuroimaging classification, where careful feature engineering and repeated cross-validation are often more decisive than increasing the depth of a network (Faiyaz et al., 2023; Dadi et al., 2019). Related comparative evidence outside neuroimaging likewise suggests that any performance gains from larger modern models should be weighed against efficiency and task constraints and that traditional machine learning methods can remain strong baselines in such settings (Zhang et al., 2026).

Prior studies in this area have typically used either conventional machine learning with handcrafted features or end-to-end deep models, whose flexibility can be difficult to manage in limited-sample settings. Our approach distinguishes itself by explicitly constructing a lightweight learned fusion framework that introduces direct anatomical coupling between tract-level evidence and connectome-level evidence. This introduces an explicit structural prior while retaining some of the representational flexibility of the learned models, which may be advantageous in limited sample settings (Thomas et al., 2019; Nenning and Langs, 2022). Related studies beyond neuroimaging have also shown that feature distillation and self-distillation can strengthen compact visual representations under constrained supervision (Huang et al., 2022; Wang et al., 2026).

### 2.2 Fusion of tract descriptors and connectome descriptors

Researchers have studied the fusion of neuroimaging features through early concatenation, modality-specific branches, attention-based aggregation, and graph-informed integration (Luo et al., 2024; Safai et al., 2022; Li et al., 2024). Related multimodal learning studies outside neuroimaging have also explored adaptive expert routing and hierarchical fusion mechanisms in sequential settings (Liu et al., 2026). A common challenge is that straightforward concatenation treats all modalities as unstructured coordinates and leaves cross-modality agreement entirely to the classifier (Jiao et al., 2024; Hangloo and Arora, 2025). For tract and connectome descriptors, this weakness is more pronounced because both modalities describe the same underlying anatomical organization at different scales (Zhang et al., 2022). A fusion framework that ignores this dependency can become statistically inefficient in the small-sample regime.

However, most generic fusion strategies reviewed in prior multimodal neuroimaging literature do not explicitly encode shared anatomical structures across imaging scales (Zhang et al., 2020; Luo et al., 2024; Li et al., 2024). In contrast to these approaches, our framework introduces an explicit alignment between tract-level and connectome-level information by using predefined anatomical families. It also preserves separate global residual paths for the two modalities so that cross-modal interactions are guided by anatomical grouping rather than learned solely from unconstrained feature concatenation.

### 2.3 Anatomical priors and consistency regularization

Researchers frequently use anatomical priors or other domain-informed constraints to improve stability in medical image analysis and neuroimaging representation learning (Dong et al., 2025; Han et al., 2024). Regularizers that enforce agreement across views, augmentations, or modalities can stabilize training and encode domain knowledge in a controlled manner (Li and Quan, 2025; Yang et al., 2025). Related studies in broader visual representation learning have likewise used student-centered distillation and post-distillation to improve supervision transfer and optimization stability (Yang et al., 2024; Bao et al., 2023). However, in very small cohorts, the effect of additional regularization can depend heavily on sample size, model choice, and how well the imposed constraint matches the underlying data structure (Jollans et al., 2019).

Many regularization strategies are generic and are not designed to reflect brain-specific anatomical structures (Dalca et al., 2018). More broadly, structured and semantically focused representation learning has demonstrated that modeling dependencies beyond direct pairwise relations can provide richer supervisory signals in transfer settings (Wang et al., 2024; Lin et al., 2025). Related studies on composed image and video retrieval further show the value of explicitly modeling entities, modification relations, directional evidence, and noisy multimodal correspondence (Li et al., 2025, 2026a,b). Our study differs by introducing a family-aligned tract-connectome consistency term that is lightweight and anatomically interpretable. This constraint is intended to encourage agreement between the tract-level and connectome-level representations within predefined anatomical families. In the present study, we evaluate this term as a mild auxiliary constraint rather than as a guaranteed source of performance improvement.

## 3 Method

**Figure 2:**
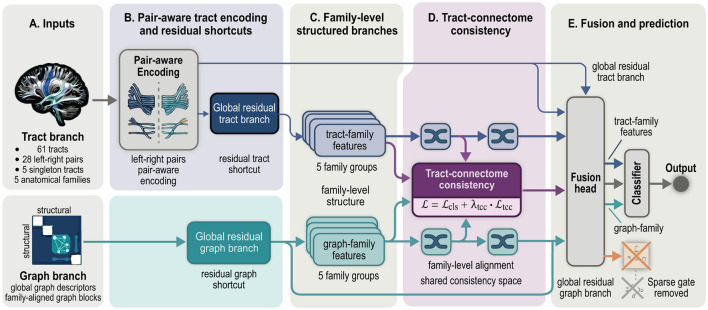
Architectural overview of the TC2-Res structured fusion network. **(A)** The model receives structural connectome graphs and tract-level features. **(B)** Tracts undergo a pair-aware bilateral summarization that maps left-right homologous pairs to side-exchange-invariant descriptors, while both modalities maintain retained global branches to preserve global modality information. **(C)** Features are processed through lightweight, family-level structured branches divided into five anatomical groups. **(D)** A tract-connectome consistency module aligns matched anatomical families in a shared latent space, driven by the combined objective function $L=L_{\text{cls}}+\lambda L_{\text{tcc}}$. **(E)** The final fusion head concatenates the retained global branches with the family-level features for prediction. As verified through ablation, sparse gating is explicitly removed to prevent instability in the small-sample regime.

Figure 2 outlines the TC2-Res architecture, and the mathematical notation, objective, regularizer, and theoretical statements are formalized in Equations 1–45.

### 3.1 Problem setup

We consider supervised classification using two anatomically related modalities. Let Ω_*t*_ denote the tract feature domain after pair-aware preprocessing, ${\text{Ω}}_{g}={\text{R}}^{d_{g,\text{glob}}}\times \prod_{f=1}^{F}{\text{R}}^{d_{g,f}}$ denote the graph feature domain, and let Ω = Ω_*t*_×Ω_*g*_ be the full input domain. The label space is Y={0,1} for the primary task. The same architecture is also evaluated in a secondary three-class setting in the Experiments section; however, the formal analysis below is presented for the primary binary task. A population distribution ℙ is defined on Ω×Y, and the observed sample is


$$\begin{array}{l} D_{n}={\left\{\left(x_{i}^{t,\text{raw}},x_{i}^{g,\text{glob}},{\left\{x_{i,f}^{g}\right\}}_{f=1}^{F},y_{i}\right)\right\}}_{i=1}^{n}. \end{array}$$ (1)

Here $x_{i}^{t,\text{raw}}$ is the raw tract descriptor before pair-aware preprocessing, $x_{i}^{g,\text{glob}}\in {\text{R}}^{d_{g,\text{glob}}}$ is the global graph descriptor, $x_{i,f}^{g}\in {\text{R}}^{d_{g,f}}$ is the family-level graph descriptor of anatomical family *f*, and *y*_*i*_ is the class label. Let *T*_pair_ denote the pair-aware preprocessing map and write ${\tilde{x}}_{i}^{t}=T_{\text{pair}}\left(x_{i}^{t,\text{raw}}\right)\in {\text{Ω}}_{t}$ for the tract input seen by the classifier. The full model input is then


$$\begin{array}{l} x_{i}=({\tilde{x}}_{i}^{t},x_{i}^{g,\text{glob}},{\left\{x_{i,f}^{g}\right\}}_{f=1}^{F})\in \text{Ω}. \end{array}$$ (2)

In the frozen dataset used in this study, *n* = 42, *F* = 5, *d*_*g*, glob_ = 154, and $\overset{F}{\underset{f=1}{\sum }}d_{g,f}=46$.

The model is a map $f_{\theta }:\text{Ω}\to \Delta^{K-1}$ with the parameter vector θ∈Θ, where Δ^*K*−1^ denotes the probability simplex and *K* = 2 for the primary task. We write the population risk and empirical risk as


$$\begin{array}{lll} R\left(\theta \right) & = & {𝔼}_{\left(X,Y\right)\sim \mathbb{P}}\ell_{\text{CE}}(Y,f_{\theta }\left(X\right)), \end{array}$$ (3)

$$\begin{array}{lll} {\hat{R}}_{n}\left(\theta \right) & = & \frac{1}{n}\overset{n}{\underset{i=1}{\sum }}\ell_{\text{CE}}(y_{i},f_{\theta }\left(x_{i}\right)). \end{array}$$ (4)

These two expressions are written first. The tract-connectome consistency term introduced later should be understood as a structural restriction on the function class used to minimize ${\hat{R}}_{n}$ rather than as a separate objective without a statistical role.

For structured fusion, the tract representation is partitioned into family blocks,


$$\begin{array}{l} {\tilde{x}}_{i}^{t}=\text{concat}\left(x_{i,1}^{t},\ldots ,x_{i,F}^{t}\right), \end{array}$$ (5)

where $x_{i,f}^{t}\in {\text{R}}^{d_{t,f}}$ is the tract subvector of family *f* and $\overset{F}{\underset{f=1}{\sum }}d_{t,f}=d_{t}$. In this dataset, the pair-aware tract dimension is *d*_*t*_ = 661. The design goal is to learn from global summaries and family-aligned local summaries simultaneously, while keeping each symbol tied to a concrete anatomical role.

### 3.2 Pair-aware tract representation

The raw tract table contains *P* left-right homologous tract pairs and singleton tracts (Yeatman et al., 2012). The first step of the method is written explicitly because the subsequent theoretical analysis depends on the distinction between symmetric and antisymmetric pair information. For a homologous pair *p*∈{1, …, *P*}, let $\ell_{i,p}\in {\text{R}}^{m_{p}}$ and $r_{i,p}\in {\text{R}}^{m_{p}}$ denote the left- and right-tract descriptors of subject *i*. We define the pair mean and pair difference by


$$\begin{array}{lll} s_{i,p} & = & \frac{\ell_{i,p}+r_{i,p}}{2}, \end{array}$$ (6)

$$\begin{array}{lll} a_{i,p} & = & \frac{\ell_{i,p}-r_{i,p}}{2}. \end{array}$$ (7)

These two variables are introduced together because they form an invertible reparameterization of the original pair,


$$\begin{array}{lll} \ell_{i,p} & = & s_{i,p}+a_{i,p}, \end{array}$$ (8)

$$\begin{array}{lll} r_{i,p} & = & s_{i,p}-a_{i,p}. \end{array}$$ (9)

The method maintains the symmetric coordinate *s*_*i, p*_ and discards the antisymmetric coordinate *a*_*i, p*_. Singleton tract features remain unchanged. The final pair-aware tract representation is therefore


$$\begin{array}{l} {\tilde{x}}_{i}^{t}=\text{concat}({\left\{s_{i,p}\right\}}_{p=1}^{P},x_{i,\text{single}}^{t}), \end{array}$$ (10)

where $x_{i,\text{single}}^{t}$ denotes concatenated singleton tract features. This expression is written because the classifier never sees the raw pair (ℓ_*i, p*_, *r*_*i, p*_) again after this point. All later tract symbols are derived from ${\tilde{x}}_{i}^{t}$.

For family-level fusion, the pair-aware tract vector is partitioned as


$$\begin{array}{l} {\tilde{x}}_{i}^{t}=\text{concat}\left(x_{i,1}^{t},\ldots ,x_{i,F}^{t}\right). \end{array}$$ (11)

Block $x_{i,f}^{t}$ is the family-specific tract input used by the family encoder of the anatomical family *f*. This notation is fixed here so that the tract family index in the method and the tract family index in the theory remain identical throughout the manuscript.

### 3.3 Structured fusion architecture with retained global branches

The proposed architecture combines direct global branches with structured family-level branches. This part is written before the consistency term because the consistency regularizer acts on the family embeddings that are produced in this section. The global tract branch is defined as


$$\begin{array}{l} u_{i}^{t}=\sigma (W_{t}{\tilde{x}}_{i}^{t}+b_{t})\in {\text{R}}^{d_{h}}, \end{array}$$ (12)

and the global graph branch is


$$\begin{array}{l} u_{i}^{g}=\sigma (W_{g}x_{i}^{g,\text{glob}}+b_{g})\in {\text{R}}^{d_{h}}, \end{array}$$ (13)

where *d*_*h*_ is the global hidden width and σ(·) is the elementwise non-linearity. In the final configuration, *d*_*h*_ = 32. These two branches are preserved because the family partition is informative but not exhaustive, and a small dataset should not force the model to explain all predictive variations through family-level interactions alone. In short, retained global branches preserve modality-wide residual information that may not be captured by family-level pathways alone.

For each anatomical family *f*, the tract family encoder and graph family encoder are


$$\begin{array}{lll} h_{i,f}^{t} & = & \phi_{f}^{t}\left(x_{i,f}^{t}\right)\in {\text{R}}^{d_{f}}, \end{array}$$ (14)

$$\begin{array}{lll} h_{i,f}^{g} & = & \phi_{f}^{g}\left(x_{i,f}^{g}\right)\in {\text{R}}^{d_{f}}, \end{array}$$ (15)

where *d*_*f*_ denotes the family-embedding dimension. These equations are required because the regularizer is not applied to raw tract blocks or raw graph blocks. It is applied to the learned family summaries $h_{i,f}^{t}$ and $h_{i,f}^{g}$, which are the anatomically aligned intermediate variables of the model.

The fused representation is defined as


$$\begin{array}{l} z_{i}=\text{concat}(u_{i}^{t},u_{i}^{g},h_{i,1}^{t},\ldots ,h_{i,F}^{t},h_{i,1}^{g},\ldots ,h_{i,F}^{g}), \end{array}$$ (16)

and the prediction head maps *z*_*i*_ to logits and probabilities through


$$\begin{array}{l} o_{i}=W_{o}z_{i}+b_{o}, \end{array}$$ (17)

$$\begin{array}{l} {\hat{p}}_{i}=\text{softmax}\left(o_{i}\right). \end{array}$$ (18)

The symbol *z*_*i*_ denotes the final fused state of subject *i*, *o*_*i*_ denotes the logit vector, and ${\hat{p}}_{i}$ denotes the predicted class distribution. The architecture is intentionally shallow because the primary challenge in this cohort is statistical fragility rather than representational insufficiency.

### 3.4 Family-aligned tract-connectome consistency

The consistency term is introduced only after the family encoders have been defined because the role of the regularizer is to compare matched anatomical families in a shared latent coordinate system. Let $P_{t}\in {\text{R}}^{d_{c}\times d_{f}}$ and $P_{g}\in {\text{R}}^{d_{c}\times d_{f}}$ be learned linear maps in a shared consistency space of dimension *d*_*c*_. The projected family embeddings are


$$\begin{array}{l} c_{i,f}^{t}=P_{t}h_{i,f}^{t}, \end{array}$$ (19)

$$\begin{array}{l} c_{i,f}^{g}=P_{g}h_{i,f}^{g}. \end{array}$$ (20)

These projected variables are written separately because the tract encoder and graph encoder do not share a coordinate system a priori. The projection step defines the coordinates in which family-level agreement becomes measurable.

To make the role of the regularizer explicit, we define the consensus component and disagreement component of each matched family pair by


$$\begin{array}{l} m_{i,f}=\frac{c_{i,f}^{t}+c_{i,f}^{g}}{2}, \end{array}$$ (21)

$$\begin{array}{l} d_{i,f}=\frac{c_{i,f}^{t}-c_{i,f}^{g}}{2}. \end{array}$$ (22)

These symbols are required because the subsequent theoretical analysis is not about generic feature shrinkage; rather it concerns the selective control of the disagreement coordinate. The projected family embeddings can be reconstructed from them as


$$\begin{array}{l} c_{i,f}^{t}=m_{i,f}+d_{i,f}, \end{array}$$ (23)

$$\begin{array}{l} c_{i,f}^{g}=m_{i,f}-d_{i,f}. \end{array}$$ (24)

The tract-connectome consistency loss is


$$\begin{array}{l} L_{\text{tcc}}=\frac{1}{nF}\overset{n}{\underset{i=1}{\sum }}\overset{F}{\underset{f=1}{\sum }}∥c_{i,f}^{t}-c_{i,f}^{g}{∥}_{2}^{2}, \end{array}$$ (25)

which can be rewritten exactly as


$$\begin{array}{l} L_{\text{tcc}}=\frac{4}{nF}\overset{n}{\underset{i=1}{\sum }}\overset{F}{\underset{f=1}{\sum }}∥d_{i,f}{∥}_{2}^{2}. \end{array}$$ (26)

This identity is central. This identity shows that the regularizer acts only on the family-level disagreement variables and does not directly penalize the consensus variables.

The classification term is


$$\begin{array}{l} L_{\text{cls}}=\frac{1}{n}\overset{n}{\underset{i=1}{\sum }}\ell_{\text{CE}}\left(y_{i},{\hat{p}}_{i}\right), \end{array}$$ (27)

and the full objective is


$$\begin{array}{l} L=L_{\text{cls}}+\lambda L_{\text{tcc}}, \end{array}$$ (28)

where λ≥0 denotes the regularization weight. In the final primary task configuration, λ = 0.2. The hyperparameter λ appears explicitly because the theoretical analysis below is built to explain the kind of statistical restriction imposed by this parameter.

### 3.5 Theoretical analysis

The theoretical analysis is designed to answer three method-specific questions. The first question is what information is removed by the pair-aware transform. The second question is what the consistency term changes in the learned representation is. The third question is why family-aligned matching is statistically significant. Accordingly, the results are presented in the same order as the corresponding design choices of the model. The results below do not claim universal accuracy improvement. They describe part of the hypothesis space that is altered by the proposed structural prior.

We first define the family-consistent empirical radius by


$$\begin{array}{l} {\hat{\varepsilon }}^{2}\left(\theta \right)=\frac{1}{nF}\overset{n}{\underset{i=1}{\sum }}\overset{F}{\underset{f=1}{\sum }}∥c_{i,f}^{t}-c_{i,f}^{g}{∥}_{2}^{2}=\frac{4}{nF}\overset{n}{\underset{i=1}{\sum }}\overset{F}{\underset{f=1}{\sum }}∥d_{i,f}{∥}_{2}^{2}. \end{array}$$ (29)

This scalar summarizes how strongly the current model disagrees across the matched tract and graph families in the training sample. For a generic input *x*∈Ω, let $c_{f}^{t}\left(x\right)$ and $c_{f}^{g}\left(x\right)$ denote the projected tract and graph embeddings of family *f*, and define


$$\begin{array}{lll} m_{f}\left(x\right) & = & \frac{c_{f}^{t}\left(x\right)+c_{f}^{g}\left(x\right)}{2}, \end{array}$$ (30)

$$\begin{array}{lll} d_{f}\left(x\right) & = & \frac{c_{f}^{t}\left(x\right)-c_{f}^{g}\left(x\right)}{2}. \end{array}$$ (31)

These function-valued symbols are generic-input counterparts of the sample quantities *m*_*i, f*_ and *d*_*i, f*_ defined above.

#### 3.5.1 Definition 1

For constants *B*_*c*_ ≥ 0, *B*_*d*_ ≥ 0, and ε ≥ 0, define the sample-dependent family-consistent linear readout class


$$\begin{array}{l} \begin{array}{l} G_{B_{c},B_{d},\varepsilon }=\{g_{\alpha ,\beta }\left(x\right)=2\overset{F}{\underset{f=1}{\sum }}\langle \alpha_{f},m_{f}\left(x\right)\rangle +2\overset{F}{\underset{f=1}{\sum }}\langle \beta_{f},d_{f}\left(x\right)\rangle \\ |\;\overset{F}{\underset{f=1}{\sum }}∥\alpha_{f}{∥}_{2}^{2}\leq B_{c}^{2},\;\overset{F}{\underset{f=1}{\sum }}∥\beta_{f}{∥}_{2}^{2}\leq B_{d}^{2},\;\frac{1}{n}\overset{n}{\underset{i=1}{\sum }}\overset{F}{\underset{f=1}{\sum }}∥d_{i,f}{∥}_{2}^{2}\leq \frac{F\varepsilon^{2}}{4}\}. \end{array} \end{array}$$ (32)

Vector α_*f*_ weights the consensus coordinates of family *f*, and vector β_*f*_ weights the disagreement coordinates of family *f*. This class is introduced because the role of the regularizer is easiest to observe after the family embeddings have been decomposed into *m*_*f*_ and *d*_*f*_.

#### 3.5.2 Proposition 1

Let *S* = (*s*_1_, …, *s*_*P*_) and *A* = (*a*_1_, …, *a*_*P*_) denote the random symmetric and antisymmetric coordinates, respectively, and let *S*_*i*_ = (*s*_*i*, 1_, …, *s*_*i, P*_) and *A*_*i*_ = (*a*_*i*, 1_, …, *a*_*i, P*_) denote their realizations for subject *i*. We assume that *Y*⊥*A*∣*S*. Then the Bayes posterior satisfies


$$\begin{array}{l} \eta^{⋆}\left(S,A\right)=\mathbb{P}\left(Y=1|S,A\right)=\mathbb{P}\left(Y=1|S\right), \end{array}$$ (33)

and each Bayes classifier can be written as a function of *S*. The proof is provided in Supplementary Appendix, Section 1.1. This result indicates that, under the stated conditional invariance assumption, the pair-aware map removes nuisance coordinates rather than label-relevant coordinates.

#### 3.5.3 Theorem 1

For any family-level linear readout


$$\begin{array}{l} q_{w}\left(x\right)=\overset{F}{\underset{f=1}{\sum }}\langle w_{f}^{t},c_{f}^{t}\left(x\right)\rangle +\overset{F}{\underset{f=1}{\sum }}\langle w_{f}^{g},c_{f}^{g}\left(x\right)\rangle , \end{array}$$ (34)

define


$$\begin{array}{lll} \alpha_{f} & = & \frac{w_{f}^{t}+w_{f}^{g}}{2}, \end{array}$$ (35)

$$\begin{array}{lll} \beta_{f} & = & \frac{w_{f}^{t}-w_{f}^{g}}{2}. \end{array}$$ (36)

Then


$$\begin{array}{l} q_{w}\left(x\right)=2\overset{F}{\underset{f=1}{\sum }}\langle \alpha_{f},m_{f}\left(x\right)\rangle +2\overset{F}{\underset{f=1}{\sum }}\langle \beta_{f},d_{f}\left(x\right)\rangle , \end{array}$$ (37)

and the consistency penalty satisfies


$$\begin{array}{l} L_{\text{tcc}}=\frac{4}{nF}\overset{n}{\underset{i=1}{\sum }}\overset{F}{\underset{f=1}{\sum }}∥d_{i,f}{∥}_{2}^{2}. \end{array}$$ (38)

Hence, the proposed regularizer penalizes only the disagreement coordinates and leaves the consensus coordinates unpenalized. The proof is provided in Supplementary Appendix, Section 1.2. This result indicates that the regularizer acts on disagreement directions rather than on all family evidence equally.

#### 3.5.4 Theorem 2

Let ℓ be a loss function that is 1-Lipschitz in its score argument and is bounded by [0, 1]. For the class $G_{B_{c},B_{d},\varepsilon }$ in Definition 1, define


$$\begin{array}{l} {\hat{M}}_{n}^{2}=\frac{1}{n}\overset{n}{\underset{i=1}{\sum }}\overset{F}{\underset{f=1}{\sum }}∥m_{i,f}{∥}_{2}^{2}. \end{array}$$ (39)

Then, for any δ∈(0, 1), with probability at least 1−δ, every $g\in G_{B_{c},B_{d},\varepsilon }$ satisfies


$$\begin{array}{l} R_{\ell }\left(g\right)\leq {\hat{R}}_{\ell ,n}\left(g\right)+\frac{4B_{c}{\hat{M}}_{n}}{\sqrt{n}}+2B_{d}\varepsilon \sqrt{\frac{F}{n}}+3\sqrt{\frac{\log\left(2/\delta \right)}{2n}}. \end{array}$$ (40)

The proof is provided in Supplementary Appendix, Section 1.3. This result indicates that the disagreement radius ε enters the generalization term directly. A smaller family disagreement budget tightens part of the bounds associated with disagreement direction.

#### 3.5.5 Definition 2

For family prototypes $\mu_{1},\ldots ,\mu_{F}\in {\text{R}}^{d_{c}}$, define the family-separation margin by


$$\begin{array}{l} {\text{Δ}}_{\min}=\underset{f\neq f^{\prime }}{\min}∥\mu_{f}-\mu_{f^{\prime }}{∥}_{2}. \end{array}$$ (41)

This quantity is introduced because the benefit of correct anatomical pairing depends on how distinct the family prototypes are in the shared consistency space. The next result formalizes the intuition that correct anatomical pairing should be preferred only when distinct families remain separated in the shared consistency space.

#### 3.5.6 Theorem 3

Assume that the projected family embeddings admit the population decomposition


$$\begin{array}{lll} C_{f}^{t} & = & \mu_{f}+\xi_{f}^{t}, \end{array}$$ (42)

$$\begin{array}{lll} C_{f}^{g} & = & \mu_{f}+\xi_{f}^{g}, \end{array}$$ (43)

where $E\xi_{f}^{t}=0$, $E\xi_{f}^{g}=0$, and the cross terms are uncorrelated. For a permutation π of {1, …, *F*}, define the population pairing risk


$$\begin{array}{l} R_{\text{pair}}\left(\pi \right)=\frac{1}{F}\overset{F}{\underset{f=1}{\sum }}𝔼∥C_{f}^{t}-C_{\pi \left(f\right)}^{g}{∥}_{2}^{2}. \end{array}$$ (44)

Then


$$\begin{array}{l} R_{\text{pair}}\left(\pi \right)=R_{\text{pair}}\left(\text{id}\right)+\frac{1}{F}\overset{F}{\underset{f=1}{\sum }}∥\mu_{f}-\mu_{\pi \left(f\right)}{∥}_{2}^{2}. \end{array}$$ (45)

Consequently, if Δ_min_>0, then the identity permutation is the unique minimizer of $R_{\text{pair}}$. The proof is provided in Supplementary Appendix, Section 1.4. This result indicates that the family index is not merely a convenient grouping device. Under the stated model, incorrect family-matching creates irreducible excess risk.

Taken together, Proposition 1 and Theorems 1 to 3 explain different parts of the method. Proposition 1 justifies pair-aware preprocessing under an explicit conditional invariance assumption. Theorem 1 shows that the consistency term acts only on the disagreement coordinates. Theorem 2 links the disagreement radius to a sample-dependent generalization term. Theorem 3 explains why family-aligned matching is a meaningful statistical prior rather than an arbitrary design choice.

### 3.6 Optimization details

The deep models are trained with stratified five-fold cross-validation under three random seeds: early stopping, learning rate 10^−3^, weight decay 10^−4^, cosine scheduling, dropout of 0, and threshold tuning for the binary task. The final selected binary configuration uses the global hidden width *d*_*h*_ = 32, structured fusion with retained global branches, pair-aware tract encoding, family-aligned graph blocks, no sparse gating, and consistency weight λ = 0.2. In addition to the standard three-seed protocol, the final binary comparison is re-evaluated with a repeated five-fold redesign over nine seeds, which yields 45 paired fold points and repeated subject-level comparison summaries. These implementation details are retained in the main text because they are directly useful for reproducing the reported experiments.

## 4 Experiment

### 4.1 Dataset and protocol

The dataset contains 42 subjects, including 17 football athletes, 16 cross-country athletes, and 9 non-athletes (Caron et al., 2021). The tract pipeline yields 61 tracts with 28 left-right homologous pairs and 5 singleton tracts (Caron et al., 2021). The graph pipeline provides two complementary graph-derived inputs: 154 global graph features for the global residual branch and 46 family-aligned graph descriptors distributed across five anatomical families for the family encoders and the tract-connectome consistency term, derived from the processed athlete dataset and reproducible neuroimaging workflows reported previously (Caron et al., 2021). Accordingly, we report branch-specific input dimensions rather than a single fused raw-feature count because the global graph features and family-aligned graph descriptors play different architectural roles. Quality control of the processed tables reports 28 missing cells in the tract and fused tables and no missing cells in the graph table. Classical baselines are evaluated by nested repeated stratified cross validation (Varoquaux et al., 2017; Varoquaux, 2018; Jollans et al., 2019). Deep models, including the learned fusion baselines and TC2-Res, use three seeds and fixed stratified five-fold splits per seed. The final binary comparison is further checked with a repeated nine-seed redesign that yields 45 paired fold evaluations. The final primary-task configuration uses a hidden width of 32, dropout of 0, consistency weight of 0.2, learning rate 10^−3^, weight decay 10^−4^, cosine scheduling, no class weighting, and threshold tuning for the binary task. Sparse gating is excluded from the final model because the matched experiments do not support its retention. Table 1 summarizes the dataset composition, input dimensions, and quality-control information.

**Table 1:** Dataset summary.

Item	Scope	Value
Subjects	Frozen cohort	42
Football athletes	Binary task positive class	17
Cross-country athletes	Three-class task	16
Non-athletes	Three-class task	9
Tract features (raw)	Before pair-aware transform	1038
Tract features (pair-aware)	Input to TC2-Res	661
Graph features (global)	Global residual branch	154
Graph features (family aligned)	Family encoders	46
Input dimensions (by branch)	Reported separately	661/154/46
Missing cells (tract/fused)	Quality control summary	28
Missing cells (graph)	Quality control summary	0

### 4.2 Baselines and evaluation targets

The main comparisons are organized into three model groups. The first group contains strong classical baselines with linear support vector machine (SVM) as the primary reference (Cortes and Vapnik, 1995). The second group contains learned baselines such as tract-only multilayer perceptrons (MLPs) and naive learned fusion by direct concatenation. The third group contains structured learned fusion variants, including matched ablations that remove the consistency term or pair-aware transform. The primary evaluation focuses on balanced accuracy, AUROC, and F1 for football vs. others (Brodersen et al., 2010; Hand and Till, 2001). The secondary evaluation focuses on macro F1, balanced accuracy, and macro AUROC for three-class recognition.

### 4.3 Primary task results

Table 2 summarizes the primary tasks. The strongest overall classifier is the linear SVM, achieving a balanced accuracy of 0.8604, AUROC of 0.9441, and F1 score of 0.8312. Among the learned models, the naive fusion baseline reaches a balanced accuracy of 0.7489, AUROC of 0.8589, and F1 score of 0.7113, whereas the final TC2-Res model achieves 0.7656, 0.8433, and 0.7276, respectively, in the verified evaluation summary. TC2-Res also attains the highest sensitivity (0.8778), while its specificity (0.6533) remains lower than that of both linear SVM and naive learned fusion.

**Table 2:** Primary task results for football vs. others.

Model	Bal. acc.	AUROC	F1	Sens.	Spec.
Linear SVM	0.8604	0.9441	0.8312	0.8630	0.8578
Naive learned fusion multilayer perceptron (MLP)	0.7489	0.8589	0.7113	0.7778	0.7200
TC2-Res	0.7656	0.8433	0.7276	0.8778	0.6533

The best, second, and third results are highlighted in yellow, orange, and blue backgrounds, respectively.

Overall, TC2-Res achieved a slightly higher mean balanced accuracy and F1 score than the naive learned fusion baseline in the primary task, but it did not surpass the strongest classical baseline. The gain should be interpreted cautiously: it was small in magnitude, heterogeneous across folds, and accompanied by lower specificity than the naive fusion baseline. Therefore, in this cohort, the main empirical conclusion is not that TC2-Res is the strongest classifier but that anatomically structured learned fusion may provide a limited aggregate advantage over naive learned fusion under severe data constraints.

### 4.4 Ablation on consistency and pair-aware encoding

The matched ablation study isolates the contributions of the tract-connectome consistency term and pair-aware tract transform. Table 3 reports the matched ablation results for the consistency term, pair-aware encoding, and sparse-gate control. Removing the consistency term reduces the balanced accuracy from 0.7656 to 0.7528 and reduces F1 from 0.7276 to 0.7124, while the AUROC remains 0.8433. Removing pair-aware encoding reduces the balanced accuracy to 0.7433, AUROC to 0.8378, and F1 to 0.7027. The sparse gate version is also weaker than the final residual model, with a balanced accuracy of 0.7506 and an F1 of 0.7140. These ablations suggest that pair-aware tract summarization contributes more consistently than the consistency term to the observed aggregate difference in the primary task. In contrast, the empirical evidence for a distinct gain attributable to the consistency term is limited, especially when compared against the matched no tract-connectome consistency (no-TCC) variant. Sparse gating is not supported by matched experiments and is therefore excluded from the final model.

**Table 3:** Matched ablation on the primary task.

Variant	Bal. acc.	AUROC	F1
TC2-Res	0.7656	0.8433	0.7276
Without consistency term	0.7528	0.8433	0.7124
Without pair-aware encoding	0.7433	0.8378	0.7027
Sparse-gate negative control	0.7506	0.8456	0.7140

The best, second, and third results are highlighted with yellow, orange, and blue backgrounds.

### 4.5 Stability and paired comparison analysis

The stability summary clarifies how primary task gains should be interpreted. Across 15 seed-fold evaluations, TC2-Res reaches a mean balanced accuracy of 0.7656 with a standard deviation of 0.1015 and a 95% confidence interval from 0.7144 to 0.8145. Relative to naive learned fusion, the mean balanced accuracy gain is 0.0167, but only 6 of the 15-fold-level comparisons are positive, and the Wilcoxon signed-rank *p*-value is 0.6368. Relative to the consistency-free variant, the mean balanced accuracy gain is 0.0128, but only 2 of 15 fold-level comparisons are positive, and the Wilcoxon *p*-value is 0.2850. The repeated 45-point redesign preserves the direction of the aggregate comparison against naive fusion, with a mean balanced accuracy delta of 0.0202 and 34 of 45 non-negative fold comparisons; however, the repeated comparison against the no-TCC model shrinks to a near-zero mean delta of 0.0020. Taken together, these findings support a restrained interpretation: TC2-Res shows a mild aggregate advantage over naive learned fusion on the primary task, but that advantage is not uniformly expressed fold-by-fold, and the evidence for improvement over the matched no-TCC variant is notably weaker. Table 4 reports the aggregated confusion matrices for TC2-Res and naive learned fusion.

**Table 4:** Primary-task confusion matrices aggregated over the verified evaluation summary.

True class	TC2-Res (predicted)		Naive fusion (predicted)	
	Class 0	Class 1	Class 0	Class 1
Class 0	**49**	26	**54**	21
Class 1	7	**44**	12	**39**

The correct predictions (the main diagonal) are highlighted. These aggregate counts are shown for interpretive comparison and are not expected to numerically match the fold-wise mean metrics in Table 2, which are averaged across evaluations.

The repeated redesign analysis follows the same direction. At the subject level, the repeated comparison against naive learned fusion gives a mean delta of 0.0212 with 15 positive subjects and 33 non-negative subjects out of 42. The repeated comparison against the no-TCC model yields a near-zero subject-level mean delta with only 3 positive subjects and 38 non-negative subjects. This repeated result reinforces the same interpretation rather than changing it. The method provides a mild aggregate benefit on the primary task against naive learned fusion; however, the magnitude and consistency of that benefit remain limited by the small-sample regime and become much weaker when the comparison is restricted to the matched no-TCC variant.

### 4.6 Secondary task and negative findings

The secondary three-class task is intentionally treated as a robustness stress test rather than a second main victory claim. The secondary-task results are summarized in Table 5. Linear SVM again provides the strongest overall result, with macro F1 0.8621, balanced accuracy 0.8685, and macro AUROC 0.9537. The strongest learned baseline is a tract-only multilayer perceptron (MLP) with macro F1 0.6812, balanced accuracy 0.7185, and macro AUROC 0.8784. The strongest learned fusion baseline reaches macro F1 0.6719, balanced accuracy 0.7056, and macro AUROC 0.8561. The best TC2 variant for this task is a model without the consistency term, which reaches macro F1 0.6642, balanced accuracy 0.7056, and macro AUROC 0.8632. Accordingly, the secondary task does not support the claim that TC2-style modeling is strongest across all settings; instead, it serves as a supplementary robustness test that helps define the boundary conditions of the primary-task inductive bias.

**Table 5:** Secondary task results.

Model	Macro F1		Bal. acc.		Macro AUROC	
	mean	sd	mean	sd	mean	sd
Tract-only MLP	0.6812	0.1668	0.7185	0.1344	0.8784	0.1003
Naive learned fusion MLP	0.6719	0.1459	0.7056	0.1223	0.8561	0.1067
Best TC2 variant	0.6642	0.2440	0.7056	0.2123	0.8632	0.1215
Linear SVM	0.8621	–	0.8685	–	0.9537	–

The best, second, and third results (for the mean) are highlighted with yellow, orange, and blue backgrounds.

This result suggests that the aggregate advantage observed in the primary binary task did not transfer robustly to the secondary three-class setting. Therefore, the same inductive bias appears to be task-dependent in this dataset, rather than uniformly beneficial across classification settings. It also supports the decision to exclude sparse gating from the final model because the more complex parameterization did not produce a reliable gain in the frozen experiments.

### 4.7 Interpretive analysis of the paired comparisons

**Figure 3:**
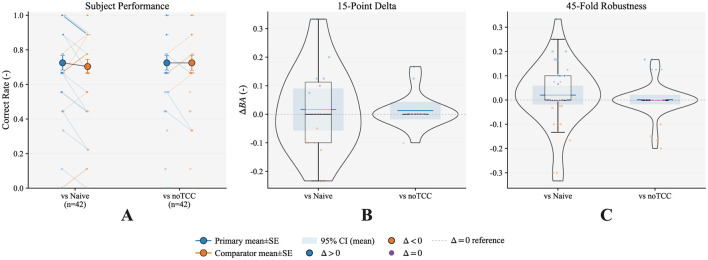
Paired experimental analysis on the primary classification task. **(A)** Subject-level correct-rate performance (*n* = 42) comparing TC2-Res with naive learned fusion and the no-TCC variant. Colored markers indicate individual subject changes (Δ > 0, Δ < 0, or Δ = 0). **(B)** Distribution of paired balanced accuracy differences (ΔBA) evaluated using a 15-point seed-fold design. **(C)** Distribution of paired balanced accuracy differences (ΔBA) evaluated using a 45-fold repeated robustness design. In panels B and C, shaded regions represent the 95% confidence intervals of the mean.

Figure 3 summarizes the paired experimental comparisons of the primary task. The subject-level panel shows that the advantage of TC2-Res over naive learned fusion is not universal across subjects, but the distribution shifts modestly toward non-negative differences. The fold-level paired comparisons show the same pattern: the average improvement is positive against naive learned fusion in both the 15-fold and repeated 45-fold designs; however, the effect is heterogeneous rather than uniform. In contrast, the comparison against the matched no-TCC variant is much weaker, indicating that the contribution of the consistency term is present but limited. Taken together with Table 6, Figure 3 supports the same quantitative interpretation: TC2-Res shows a modest aggregate advantage over naive learned fusion on the primary task, but the gain remains variable across folds and subjects.

**Table 6:** Paired comparison summary on the primary task.

Comparison	Design	Mean Δ bal. acc.	Positive	Non-negative	Wilcoxon *p*
TC2-Res vs. naive fusion	15-fold	0.0167	6/15	–	0.6368
TC2-Res vs. no-TCC variant	15-fold	0.0128	2/15	–	0.2850
TC2-Res vs. naive fusion	45-fold repeated	0.0202	19/45	34/45	–
TC2-Res vs. no-TCC variant	45-fold repeated	0.0020	5/45	41/45	–

These visual summaries are consistent with the quantitative results but should not be overinterpreted. They support a small aggregate shift in favor of TC2-Res relative to naive learned fusion on the primary task, while also showing that the effect is heterogeneous and that the corresponding evidence against the matched no-TCC variant is much weaker.

## 5 Conclusion

This paper presents TC2-Res, a lightweight structured fusion framework for tract-level and connectome-level brain imaging in a small-sample athlete cohort. This method combines pair-aware tract summarization, retained global modality branches, family-level encoders, and a family-aligned tract-connectome consistency term. In the primary football-vs.-others task, TC2-Res achieved a small positive mean difference relative to a matched naive learned fusion baseline, but it did not surpass the strongest classical linear baseline, and the observed gain was heterogeneous across folds. Empirical evidence for a distinct contribution from the consistency term was limited, particularly in comparison with the matched no-TCC variant. In the secondary three-class task, the TC2-style models did not provide the strongest learned result. Overall, the findings support TC2-Res as a lightweight structured fusion design whose empirical benefit in this cohort is modest and preliminary rather than uniform or definitive. Future studies should evaluate whether the same structural prior could become more beneficial in larger cohorts under external validation or with richer graph representations.

## Data Availability

The datasets presented in this study can be found in online repositories. The names of the repository/repositories and accession number(s) can be found below: https://brainlife.io/pub/5f2c3765beafe924c962dd8d.
