# Supplementary material for: TC2-Res: a structured fusion of tract-level and connectome-level brain imaging in small-sample cohorts of athletes
Source: Front Neuroanat. 2026 Jun 3;20:1841420. doi: 10.3389/fnana.2026.1841420 (PMC13272116; doi:10.3389/fnana.2026.1841420)
Supplement: Supplementary file 1 [file Supplementary_file_1.pdf]

1 PROOFS FOR THE THEORETICAL ANALYSIS

1.1 Proof of Proposition 1

Let $\eta^\star(S, A) = \mathbb{P}(Y = 1 \mid S, A)$ denote the Bayes posterior under the raw paired tract coordinates. By the assumption $Y \perp A \mid S$, one has

$$\mathbb{P}(Y = 1 \mid S, A) = \mathbb{P}(Y = 1 \mid S).$$

Therefore

$$\eta^\star(S, A) = \eta^\star(S).$$

A Bayes classifier is obtained by thresholding the posterior at $1/2$, hence

$$g^\star(S, A) = \mathbf{1}\big[\eta^\star(S, A) \geq 1/2\big] = \mathbf{1}\big[\eta^\star(S) \geq 1/2\big].$$

Thus every Bayes classifier can be written as a function of S alone. This completes the proof.

This proof shows that the pair-aware transform is justified whenever the discarded antisymmetric coordinate is conditionally irrelevant once the symmetric coordinate is known.

1.2 Proof of Theorem 1

By the definitions of α_f and β_f,

$$w_f^t = \alpha_f + \beta_f,$$
$$w_f^g = \alpha_f - \beta_f.$$

Using $c_f^t = m_f + d_f$ and $c_f^g = m_f - d_f$, one obtains

$$\langle w_f^t, c_f^t \rangle + \langle w_f^g, c_f^g \rangle = \langle \alpha_f + \beta_f, m_f + d_f \rangle + \langle \alpha_f - \beta_f, m_f - d_f \rangle.$$

Expanding the inner products gives

$$\langle w_f^t, c_f^t \rangle + \langle w_f^g, c_f^g \rangle = 2\langle \alpha_f, m_f \rangle + 2\langle \beta_f, d_f \rangle.$$

Summing over f yields

$$q_w(x) = 2\sum_{f=1}^{F} \langle \alpha_f, m_f(x) \rangle + 2\sum_{f=1}^{F} \langle \beta_f, d_f(x) \rangle.$$

For the regularizer, the definition of $d_{i,f}$ gives

$$c_{i,f}^t - c_{i,f}^g = 2d_{i,f}.$$

Taking squared Euclidean norms gives

$$\|c_{i,f}^t - c_{i,f}^g\|_2^2 = 4\|d_{i,f}\|_2^2.$$

Summing over subjects and families yields

$$\mathcal{L}_{\text{tcc}} = \frac{4}{nF}\sum_{i=1}^{n}\sum_{f=1}^{F}\|d_{i,f}\|_2^2.$$

Thus the consistency penalty depends only on disagreement coordinates and is independent of the consensus coordinates $m_{i,f}$. This completes the proof.

This proof shows that the regularizer acts only along disagreement directions in the consensus and disagreement basis induced by the method.

1.3 Proof of Theorem 2

For each sample i, define the stacked consensus vector and stacked disagreement vector by

$$M_i = \text{concat}(m_{i,1}, \ldots, m_{i,F}),$$
$$D_i = \text{concat}(d_{i,1}, \ldots, d_{i,F}).$$

Likewise define the stacked coefficient vectors by

$$\alpha = \text{concat}(\alpha_1, \ldots, \alpha_F),$$
$$\beta = \text{concat}(\beta_1, \ldots, \beta_F).$$

Then every $g \in \mathcal{G}_{B_c,B_d,\varepsilon}$ can be written as

$$g(x_i) = 2\langle \alpha, M_i\rangle + 2\langle \beta, D_i\rangle,$$

with $\|\alpha\|_2 \le B_c$ and $\|\beta\|_2 \le B_d$. The empirical Rademacher complexity therefore satisfies

$$\widehat{\mathfrak{R}}_n(\mathcal{G}_{B_c,B_d,\varepsilon}) = \frac{1}{n}\mathbb{E}_\sigma \sup_{g\in\mathcal{G}_{B_c,B_d,\varepsilon}} \sum_{i=1}^{n}\sigma_i g(x_i),$$

where $\sigma_1, \ldots, \sigma_n$ are independent Rademacher variables. By the representation above,

$$\widehat{\mathfrak{R}}_n(\mathcal{G}_{B_c,B_d,\varepsilon}) \le \frac{2}{n}\mathbb{E}_\sigma \sup_{\|\alpha\|_2\le B_c}\Big\langle \alpha, \sum_{i=1}^{n}\sigma_i M_i\Big\rangle + \frac{2}{n}\mathbb{E}_\sigma \sup_{\|\beta\|_2\le B_d}\Big\langle \beta, \sum_{i=1}^{n}\sigma_i D_i\Big\rangle.$$

Applying Cauchy gives

$$\widehat{\mathfrak{R}}_n(\mathcal{G}_{B_c,B_d,\varepsilon}) \le \frac{2B_c}{n}\mathbb{E}_\sigma\Big\|\sum_{i=1}^{n}\sigma_i M_i\Big\|_2 + \frac{2B_d}{n}\mathbb{E}_\sigma\Big\|\sum_{i=1}^{n}\sigma_i D_i\Big\|_2.$$

Jensen and the Khintchine identity yield

$$\mathbb{E}_\sigma\Big\|\sum_{i=1}^{n}\sigma_i M_i\Big\|_2 \le \left(\sum_{i=1}^{n}\|M_i\|_2^2\right)^{1/2},$$

$$\mathbb{E}_\sigma \Big\| \sum_{i=1}^n \sigma_i D_i \Big\|_2 \le \left(\sum_{i=1}^n \|D_i\|_2^2 \right)^{1/2}.$$

Hence

$$\widehat{\mathfrak{R}}_n(\mathcal{G}_{B_c,B_d,\varepsilon}) \le \frac{2B_c}{n} \left(\sum_{i=1}^n \|M_i\|_2^2 \right)^{1/2} + \frac{2B_d}{n} \left(\sum_{i=1}^n \|D_i\|_2^2 \right)^{1/2}.$$

By definition of $\widehat{M}_n$,

$$\frac{2B_c}{n} \left(\sum_{i=1}^n \|M_i\|_2^2 \right)^{1/2} = \frac{2B_c \widehat{M}_n}{\sqrt{n}}.$$

Also, the constraint in Definition 1 implies

$$\frac{1}{n} \sum_{i=1}^n \|D_i\|_2^2 = \frac{1}{n} \sum_{i=1}^n \sum_{f=1}^F \|d_{i,f}\|_2^2 \le \frac{F\varepsilon^2}{4}.$$

Therefore

$$\frac{2B_d}{n} \left(\sum_{i=1}^n \|D_i\|_2^2 \right)^{1/2} \le B_d \varepsilon \sqrt{\frac{F}{n}}.$$

Combining the two terms gives

$$\widehat{\mathfrak{R}}_n(\mathcal{G}_{B_c,B_d,\varepsilon}) \le \frac{2B_c \widehat{M}_n}{\sqrt{n}} + B_d \varepsilon \sqrt{\frac{F}{n}}.$$

Since ℓ is 1-Lipschitz and bounded in $[0, 1]$, a standard Rademacher generalization bound implies that, with probability at least $1 - \delta$, every $g \in \mathcal{G}_{B_c,B_d,\varepsilon}$ satisfies

$$R_\ell(g) \le \widehat{R}_{\ell,n}(g) + 2\widehat{\mathfrak{R}}_n(\mathcal{G}_{B_c,B_d,\varepsilon}) + 3\sqrt{\frac{\log(2/\delta)}{2n}}.$$

Substituting the previous estimate yields

$$R_\ell(g) \le \widehat{R}_{\ell,n}(g) + \frac{4B_c \widehat{M}_n}{\sqrt{n}} + 2B_d \varepsilon \sqrt{\frac{F}{n}} + 3\sqrt{\frac{\log(2/\delta)}{2n}}.$$

This completes the proof.

This proof shows that the consistency radius enters the generalization term in a direct and quantitatively explicit way.

1.4 Proof of Theorem 3

Fix a permutation π of $\{1, \ldots, F\}$. By the assumed decomposition,

$$C_f^t - C_{\pi(f)}^g = (\mu_f - \mu_{\pi(f)}) + (\xi_f^t - \xi_{\pi(f)}^g).$$

Taking squared norms and expectations gives

$$\mathbb{E}\,\|C_f^t - C_{\pi(f)}^g\|_2^2 = \|\mu_f - \mu_{\pi(f)}\|_2^2 + \mathbb{E}\,\|\xi_f^t - \xi_{\pi(f)}^g\|_2^2 + 2\,\mathbb{E}\,\langle \mu_f - \mu_{\pi(f)}, \xi_f^t - \xi_{\pi(f)}^g \rangle.$$

The last term is zero because the noise terms have zero mean. Hence

$$\mathbb{E}\,\|C_f^t - C_{\pi(f)}^g\|_2^2 = \|\mu_f - \mu_{\pi(f)}\|_2^2 + \mathbb{E}\,\|\xi_f^t\|_2^2 + \mathbb{E}\,\|\xi_{\pi(f)}^g\|_2^2 - 2\,\mathbb{E}\,\langle \xi_f^t, \xi_{\pi(f)}^g \rangle.$$

By the assumed lack of cross correlation, the inner product term vanishes, so

$$\mathbb{E}\,\|C_f^t - C_{\pi(f)}^g\|_2^2 = \|\mu_f - \mu_{\pi(f)}\|_2^2 + \mathbb{E}\,\|\xi_f^t\|_2^2 + \mathbb{E}\,\|\xi_{\pi(f)}^g\|_2^2.$$

Averaging over f yields

$$\mathcal{R}_{\text{pair}}(\pi) = \frac{1}{F}\sum_{f=1}^{F}\|\mu_f - \mu_{\pi(f)}\|_2^2 + \frac{1}{F}\sum_{f=1}^{F}\mathbb{E}\,\|\xi_f^t\|_2^2 + \frac{1}{F}\sum_{f=1}^{F}\mathbb{E}\,\|\xi_{\pi(f)}^g\|_2^2.$$

Because π is a permutation,

$$\sum_{f=1}^{F}\mathbb{E}\,\|\xi_{\pi(f)}^g\|_2^2 = \sum_{f=1}^{F}\mathbb{E}\,\|\xi_f^g\|_2^2.$$

Therefore

$$\mathcal{R}_{\text{pair}}(\pi) = \frac{1}{F}\sum_{f=1}^{F}\|\mu_f - \mu_{\pi(f)}\|_2^2 + \frac{1}{F}\sum_{f=1}^{F}\mathbb{E}\,\|\xi_f^t\|_2^2 + \frac{1}{F}\sum_{f=1}^{F}\mathbb{E}\,\|\xi_f^g\|_2^2.$$

For the identity permutation,

$$\mathcal{R}_{\text{pair}}(\text{id}) = \frac{1}{F}\sum_{f=1}^{F}\mathbb{E}\,\|\xi_f^t\|_2^2 + \frac{1}{F}\sum_{f=1}^{F}\mathbb{E}\,\|\xi_f^g\|_2^2.$$

Subtracting the two displays gives

$$\mathcal{R}_{\text{pair}}(\pi) - \mathcal{R}_{\text{pair}}(\text{id}) = \frac{1}{F}\sum_{f=1}^{F}\|\mu_f - \mu_{\pi(f)}\|_2^2.$$

If $\Delta_{\text{min}} > 0$ and $\pi \neq \text{id}$, then at least one term on the right-hand side is strictly positive, so

$$\mathcal{R}_{\text{pair}}(\pi) > \mathcal{R}_{\text{pair}}(\text{id}).$$

Hence the identity permutation is the unique minimizer. This completes the proof.

This proof shows that the anatomical family index has statistical content when the family prototypes are distinct in the shared consistency space.
